# Loneliness as a Predictor of Cognitive Decline in Older Adults: Insights from a Measurement Burst Design Using the Einstein Aging Study

**DOI:** 10.1002/alz.091298

**Published:** 2025-01-09

**Authors:** Jee Eun Kang, Martin J. Sliwinski, Zita Oravecz, Christopher G. Engeland, Mindy J. Katz, Jennifer E. Graham‐Engeland

**Affiliations:** ^1^ The Pennsylvania State University, University Park, PA USA; ^2^ Pennsylvania State University, State College, PA USA; ^3^ Albert Einstein College of Medicine, Bronx, NY USA

## Abstract

**Background:**

Loneliness is linked with risk for cognitive decline and dementia among older adults, but the degree to which it predicts future risk is unclear. To investigate if loneliness acts as a predictor of cognitive decline, this study employed a measurement burst design using data from the Einstein Aging Study, where loneliness and cognition were repeatedly assessed daily, for several days, across several years. In this type of data, a major challenge to detecting subtle cognitive changes is the presence of retest/practice effects. We employed a novel process modeling approach (Bayesian Double Exponential Model; BDEM) to distinguish retest learning effects from genuine longitudinal cognitive changes.

**Method:**

Participants (n = 313; English‐speaking, community‐residing adults aged ≥70) underwent up to six waves of annual assessments. Each wave included a 16‐day ambulatory ecological momentary assessment (EMA) burst on mobile phones. Ambulatory assessments consisted of self‐report (including a question about feeling lonely now) and cognitive tests, conducted multiple times daily. This study analyzed the spatial working memory task.

Cognitive parameters capturing retest learning rate, peak performance (disentangled from retest learning) and long‐term changes in peak performance were estimated simultaneously using the BDEM. Regression analyses examined associations between mean and standard deviation (SD) of EMA loneliness at baseline and cognitive markers, controlling for demographics and mild cognitive impairment (MCI) status at baseline.

**Result:**

A higher mean and a higher variability (SD) of EMA loneliness at baseline predicted higher baseline asymptotic (peak) performance, suggesting worse peak cognitive performance at baseline. However, neither mean level nor variability of EMA loneliness at baseline predicted changes in peak performance after baseline. Notably, a higher variability of EMA loneliness was also associated with slower learning rates from repeated testing across bursts.

**Conclusion:**

This study reveals that the level and variability of loneliness in daily life, measured in near‐real time, predict concurrent peak performance and long‐term learning rates in working memory among older adults. Results, particularly regarding loneliness variability, suggest that the experience of loneliness has implications for long‐term learning rates. Not only the mean level of loneliness, but also variations in loneliness, have separate predictive value for cognitive performance over time.

